# Withdrawing or withholding treatments in health care rationing: an interview study on ethical views and implications

**DOI:** 10.1186/s12910-022-00805-9

**Published:** 2022-06-24

**Authors:** Liam Strand, Lars Sandman, Gustav Tinghög, Ann-Charlotte Nedlund

**Affiliations:** 1 Swedish National Centre for Priorities in Health, Department of Health, Medicine, and Caring Sciences, Linköping University, Sandbäcksgatan 7, 581 83 Linköping, Sweden; 2 Department of Management and Engineering, Linköping University, Linköping, Sweden

**Keywords:** Reimbursement, Disinvestment, Qualitative research, Priority setting, Equivalence thesis, Sweden

## Abstract

**Background:**

When rationing health care, a commonly held view among ethicists is that there is no ethical difference between withdrawing or withholding medical treatments. In reality, this view does not generally seem to be supported by practicians nor in legislation practices, by for example adding a ‘grandfather clause’ when rejecting a new treatment for lacking cost-effectiveness. Due to this discrepancy, our objective was to explore physicians’ and patient organization representatives’ experiences- and perceptions of withdrawing and withholding treatments in rationing situations of relative scarcity.

**Methods:**

Fourteen semi-structured interviews were conducted in Sweden with physicians and patient organization representatives, thematic analysis was used.

**Results:**

Participants commonly express internally inconsistent views regarding if withdrawing or withholding medical treatments should be deemed as ethically equivalent. Participants express that in terms of patients’ need for treatment (e.g., the treatment’s effectiveness and the patient’s medical condition) withholding and withdrawing should be deemed ethically equivalent. However, in terms of prognostic differences, and the patient-physician relation and communication, there is a clear discrepancy which carry a moral significance and ultimately makes withdrawing psychologically difficult for both physicians and patients, and politically difficult for policy makers.

**Conclusions:**

We conclude that the distinction between withdrawing and withholding treatment as unified concepts is a simplification of a more complex situation, where different factors related differently to these two concepts. Following this, possible policy solutions are discussed for how to resolve this experienced moral difference by practitioners and ease withdrawing treatments due to health care rationing. Such solutions could be to have agreements between the physician and patient about potential future treatment withdrawals, to evaluate the treatment’s effect, and to provide guidelines on a national level.

## Introduction

As more new and expensive treatments emerge, policymakers will have to assess how to prioritize treatments within a publicly funded healthcare system which is already constrained by financial scarcity. They must then ration the available healthcare resources, with disinvestment being one possibility. Disinvestments can be done either passively (e.g., by manufacturers leaving the market) or by an active policy decision, which ultimately leads to “the full withdrawal, retraction, restriction or substitution of resources from certain healthcare interventions” [1]. An active disinvestment would therefore affect the accessibility of these treatments in practice [2–4], with clinicians having to decide how to handle patients who need the treatment. Such decisions can essentially take one of two forms: withdrawing—the removal of a treatment that has been started and might be effective for that patient (but not cost-effective and therefore disinvested)—and withholding—the decision not to start a treatment intervention for a patient who will potentially benefit from it. In practice, there are examples where these two strategies are differentiated between, such as when the National Institute for Health and Care Excellence (NICE) considers a new drug treatment but deems it to lack cost-effectiveness, deciding that new patients should not be offered the drug while patients with previous access to the treatment are unaffected (see [5]). In the present study, there will be a focus on the experienced difference between withdrawing and withholding treatments in a healthcare rationing setting.

Studies exploring attitudes toward withholding and withdrawing treatments have shown that people react differently and view the two decisions as ethically [6, 7] and psychologically [8, 9] different. The differences were reportedly caused by psychological discomfort, uncertainty about laws, guidelines, and ethics, fear of sanctions [7], and religious beliefs [6, 8]. However, this previous research comes from the end-of-life care setting, where healthcare rationing is necessary since the treatment cannot be given to all patients, due to a limited number of ventilators, hospitals beds, organ donators etc., and acute consequences (the patient might die immediately)—what is termed absolute scarcity [10–13]. In disinvestment, the motivation for health rationing is financial scarcity. The main difference is that a treatment should not be given to patients since it lacks cost-effectiveness. It is technically possible to keep providing it to patients—albeit at the opportunity cost to other patients in the system—which is termed relative scarcity [10, 11, 14]. Studies from the disinvestment literature have found that the public prefers cost-ineffective treatments not being withdrawn from patients with previous access [1, 15] and effective treatments being reimbursed (and used in practice) compared to equally effective treatments that are currently neither reimbursed nor used in practice [16]. However, the responses revealed a clear case of preference reversal; the respondents had previously stated that the reimbursement status is not important, but it still affected their decision-making [16]. Thus, preferences toward withholding and withdrawing treatments seem to be malleable, depending on how scenarios are presented. This malleability is problematic, as previous studies have not managed to explain in depth why differences are experienced between withdrawing and withholding treatment.

In contrast to the empirically grounded studies, normative literature has tried to explain the differences between withdrawing and withholding treatments, mostly finding the two decisions to be ethically equivalent and that non-equivalence is a case of irrational behaviors caused largely by psychological biases. Suggested biases include withdrawal aversion, a preference for withholding treatments [17], or the sunk cost bias, a tendency for people to keep investing in a treatment because they have already invested in it [18]. It is also argued that since the consequences will be the same when withdrawing and withholding a treatment, there is an ethical equivalence between the two decisions [19]. A solution based on non-equivalence (like NICE’s solution) is criticized, since it implies that that patients with the same clinical need for a treatment would be treated differently [20, 21]. However, opponents of equivalence suggest that there is an essential ethical difference between active and passive acts [22, 23], and that withdrawing a treatment is different because it typically involves breaking a promise and a bond of trust between the physician and the patient [22, 24]. Therefore, it is argued that policymakers should differentiate between withdrawing and withholding treatments when rationing healthcare. As the normatively grounded research is rather inconclusive on why withdrawing and withholding are experienced as different and how the issue should be handled in practice, it would be valuable to consider the views of those who make and are affected by these decisions in practice.

The active disinvestment decision to withdraw treatments due to a lack of cost-effectiveness has proven to be a delicate issue [25]. If policymakers are to make active disinvestment decisions to cope with the increased financial scarcity caused by increased costs for new treatments, then it is important to have public support for such decisions [2, 26]. Unfortunately, previous research has indicated that the public does not support treatments being withdrawn due to relative scarcity.

Although we have some insight into why people experience withdrawing and withholding treatments as ethically different, it is necessary to broaden our knowledge and investigate the views of physicians and patient organizations—those who make and are affected by these decisions. A better understanding of why withdrawing and withholding treatments are experienced as ethically different could offer better advice for policymakers when formulating disinvestment policies, and could improve the ethical discussion by describing factors which might not have been considered previously. Therefore, our aim was to interview physicians and patient organization representatives to explore their experiences and perceptions of withdrawing and withholding treatments in rationing situations due to relative scarcity.

### Reimbursement of treatments in a Swedish context

In Sweden, reimbursement decisions on new treatments are usually made by the healthcare regional authorities. However, reimbursement decisions on prescription drugs are made by a national healthcare authority, the Dental and Pharmaceutical Benefits Agency (TLV), and a joint regional body at national level, the New Therapies Council, makes recommendations on other new and costly drugs. The regions have largely decided to follow these recommendations in their reimbursement decisions [27]. At the same time, Swedish physicians have a free right of prescription and can decide not to follow regional or national decisions [28]. Hence, the relationship between the national level (the state and its agencies), the regional level, and the physician level is somewhat vague concerning setting priorities for drugs [29]. Moreover, it has been enshrined in law since 1997 that decisions on reimbursement should take the human value, patient needs, and cost-effectiveness of into account, regardless of the level at which a priority decision is made [30].

## Methods

Fourteen semi-structured interviews were conducted with eight physicians and six patient organization representatives (PORs) from Sweden. The sampling of the participants were purposive [31] as we chose physicians working in healthcare areas with high influx of technology (oncology, hematology, neurology, and rare diseases) and PORs representing patients within these areas, and thus likely to have experience of withdrawing or withholding treatments. The 14 participants were on average 57 years old (min 43, max 74), 50% were males, and had educational levels ranging from secondary school to master’s degrees. In addition, 2 potential participants dropped out due to illness, 2 were not available for interview and were therefore excluded, 6 did not reply to the invitation, and 1 refused to participate as they viewed themselves as not fit for the study. Topic guides were developed with key themes, such as experiences of reimbursement decisions, which factors and arguments affect decisions, and the relationship between the physician and the patient when prioritizing treatments. The topic guides were pilot tested with one interview for each group, and were then revised. The topic guides were flexible and allowed for follow-up questions to obtain a deeper understanding of the interviewees’ experiences. Full topic guides can be accessed by contacting the corresponding author.

The interviews were conducted online via Zoom by the first author (A1) between September 2020 and May 2021. The interviews were conducted in Swedish, and all the quotations used in the text have been translated from Swedish into English. They took approximately one hour and were audio recorded and then transcribed and pseudonymized to ensure confidentiality. The physicians are referred to as PHY and patient organization representatives as POR, followed by a number to distinguish between them. A1 took field notes during the interviews and summarized them directly afterwards to capture the immediate impressions. The data was regarded as saturated by the authors when the field notes from the last interview had not given any new major insights.

The data was analyzed following the thematic framework method [31]. The transcripts were first read several times by A1 and then labelled using first-order codes close to the participants’ terms. These codes were thereafter sorted into more general themes, with the process being followed iteratively by discussion between A1 and A4. In the next step, empirical statements from each theme were identified. These statements follow what Ritchie and Lewis [31] refer to as explanatory accounts, as analyzing the codes and themes involve detecting patterns in the generated data and the statements are the assigned meaning of the generated data. The coding, thematization, and empirical statements were discussed iteratively among all co-authors, who have previous experience of qualitative methods, politics and policy analysis, ethics, psychology, and economics, to triangulate the interpretation of the generated data and decrease the risk of one-sidedness and distortions [32]. The coding was then revised, and the process was repeated until the authors agreed on the identified themes and statements. Microsoft Excel was used to manage the data.

## Findings

We identified eight different themes, encompassing individual patients’ benefit from the treatments, the relationship and communication between and among patients and physicians, and more systemic concepts such as healthcare responsibility and ethical values (see Table 1). Within these themes, we also identified 55 different empirical statements which are presented in full in Table 2.

**Table 1:** The identified themes and descriptions

Theme	Description
Patients’ need for treatment	How the patient’s need for a treatment affects decisions to withdraw or withhold treatments
Treatment effect in relation to alternative treatments	How the treatment’s effects affect withdrawing and withholding treatments
Patient–professional communication	How communication between patients and professionals affects withdrawing and withholding treatments
Patient–professional relationship	How relational factors between the patient and physician affect withdrawing and withholding treatments
Healthcare responsibility	The responsibilities of the healthcare system and its physicians when withdrawing and withholding treatments
Ethical values	Ethical values and their relative importance when withdrawing and withholding treatments
Professional support	The need for and attributes of supporting tools for physicians when withdrawing and withholding treatments
Reimbursement system	Factors which describe the context in which decisions are made about withdrawing and withholding treatments

**Table 2:** The identified statements

Themes	#	Statement	Context
Patients’ need for treatment	1	An ill medical condition can make a physician’s decision to withdraw or withhold a treatment easier	PHY/POR
	2	An ill medical condition can make the physician more willing to take higher risks and not withhold treatments	PHY
	3	It might be easier for both the physician and the patient when withdrawing or withholding a treatment from a patient if alternative treatments exist	PHY/POR
	4	The patient’s quality of life is important when deciding to withdraw or withhold treatment	PHY/POR
Treatment effect in relation to alternative treatments	5	The healthcare sector provides inefficient treatments to patients	PHY/POR
	6	Treatments are commonly withdrawn too late from patients in practice	PHY/POR
	7	Physicians sometimes withhold treatments from patients due to cost-effectiveness	PHY/POR
	8	Physicians sometimes withdraw treatments from patients due to cost-effectiveness	PHY/POR
	9	Physicians commonly withdraw treatments from patients because they are ineffective or cause harm, rather than for cost-effectiveness reasons	PHY/POR
	10	A treatment that has been proven to be ineffective for a specific patient should be withdrawn	PHY/POR
	11	It must be acceptable for physicians to withdraw ineffective treatments	PHY/POR
	12	A treatment that has proven to be effective for a specific patient should not be withdrawn by the healthcare service, even if it is not reimbursed	PHY/POR
	13	A treatment that has proven to be effective for a patient participating in a clinical study should not be withdrawn	PHY/POR
	14	Patients can understand if a treatment is withdrawn after a clinical study	POR
	15	The expected net patient benefit of a treatment can affect the physician’s decision to withdraw or withhold a treatment	PHY
	16	It can be helpful for a physician to evaluate a treatment’s effects when deciding to withdraw a treatment	PHY/POR
	17	It can be difficult for a physician to evaluate all effects a treatment has or will have for a patient	PHY/POR
	18	The use of one treatment can exclude the use of alternative treatments	POR
Patient–professional communication	19	Involving patients in decision-making can facilitate withdrawals	PHY/POR
	20	Agreements between a physician and a patient can facilitate treatment withdrawals	PHY/POR
	21	Agreements between a physician and a patient can be the difference between withdrawing and withholding treatments	POR
	22	It can be easier to withdraw a treatment if the physician informs the patient of the conditions for the treatment before starting it	PHY/POR
	23	It is easier for the physician to withdraw treatments if the patient understands the information given to them	PHY/POR
Patient–professional relationship	24	The physician should represent the patient when deciding to withdraw or withhold treatments	PHY/POR
	25	Having a relationship between the physician and the patient can facilitate treatment withdrawal	PHY/POR
	26	Spending extra time to support a patient psychologically makes it easier for the patient if their treatment is withdrawn	POR
	27	It can be comforting for relatives if the physician decides whether a treatment is withdrawn or withheld	PHY
	28	Having too close a relationship between the physician and the patient can make the physician act unprofessionally when withdrawing a treatment	PHY
	29	The physical meeting with patients makes it more difficult for physicians to decide to withdraw or withhold treatments for specific patients than for patient groups	PHY/POR
Healthcare responsibility	30	It is a physician’s obligation to withdraw ineffective or harmful treatments	PHY/POR
	31	A physician has more obligations when prescribing unofficial treatments to patients	PHY/POR
	32	Patients might lose confidence in the healthcare system if effective treatments are withdrawn because of reimbursement status	PHY
	33	Expensive treatments should be publicly funded	POR
	34	The pharmaceutical company should finance effective treatments for patients after a study is completed until an official recommendation is given	PHY/POR
Ethical values	35	It is psychologically easier to withhold a treatment due to cost-effectiveness than to withdraw it	PHY/POR
	36	There is an ethical difference between withdrawing and withholding treatments due to a lack of cost-effectiveness	PHY/POR
	37	It is more important for physicians to make an individual assessment for patients with previous access to treatments that lack cost-effectiveness than to withdraw treatments to uphold patient equality	PHY/POR
	38	Patients might not experience the same human value if their treatments are withdrawn due to a lack of cost-effectiveness	POR
	39	Withdrawing and withholding treatments differently might lead to patients seeking healthcare from other healthcare providers	PHY/POR
	40	It is unjust when different healthcare providers withdraw and withhold treatments unequally	PHY/POR
Professional support	41	Physicians feel alone when deciding to withdraw or withhold treatments	PHY
	42	It can be helpful for a physician to consult other physicians when deciding to withdraw or withhold treatments	PHY
	43	Guidelines from a national level on treating new patients and patients with previous access to treatments after new recommendations can facilitate treatment withdrawals for physicians and patients	PHY/POR
	44	Guidelines from a national level should be accessible for physicians	PHY
	45	Guidelines from a national level may not be applicable in all healthcare scenarios	POR
	46	It could be helpful for a physician to have reflected on ethical problems related to priorities when making priority decisions	PHY/POR
Reimbursement system	47	Physicians and patient organization representatives are supportive of healthcare making priority decisions	PHY/POR
	48	Physicians tend to prioritize their own patient groups	PHY
	49	Patient organizations represent their own patient groups	POR
	50	The treatment assessment process is not sufficiently transparent for patients	POR
	51	Patients are not sufficiently involved in the treatment assessment process	POR
	52	Patients want access to new treatments	POR
	53	It takes a long time for authorities to implement new treatments	PHY/POR
	54	A patient cannot demand access to the experimental treatment in a clinical study	PHY/POR
	55	There is a difference between what is medically best and what is practically possible when prioritizing treatments between patients	PHY/POR

PHY, physician; POR, patient organization representative

### Patients’ need for treatment

This theme describes how a patient’s need for a treatment affects a decision to withdraw or withhold treatments. The patient’s medical condition was described by the physicians as a “guiding star” in everything they do, and that they will withhold a treatment if they assess the patient’s “performance status” to be too poor. Similarly, they said that they would withdraw treatment if the patient no longer copes with it. A distinction was made when a patient was very ill. The physicians explained that they become more experimental and willing to take a greater risk when there is a high risk that the patient could die, or when there is a chance of long-term survival, and are therefore less willing to withhold treatments, but not “at any cost”. Furthermore, both physicians and PORs expressed that it might be easier for both the physician and the patient when withdrawing or withholding a treatment from a patient if alternative treatments exist (statement 3). This was described as making it “emotionally easier”, and that withdrawing without providing an alternative would be like “throwing someone into an abyss”.

“It makes it much easier when an alternative treatment exists. And this very agreement, that “We will give you the best possible care, the best possible treatment”. And, say, “We will offer the best possible treatment, but the cost is always an aspect for us because we live in a reality, which means that we really want to switch from this to that now and we believe it will work fine. Sure, there is a risk of side-effects, and we might see some worse effect.” But that is kind of compared to “You will receive the best possible treatment, but you do not get this, you get nothing instead”. That’s kind of throwing someone into an abyss.” (POR1)

The physicians highlighted that the patient’s quality of life is important when deciding to withdraw or withhold treatments, as is considering the patient’s wishes. An example was given of not withholding a treatment so that a mother in her thirties would get a few months extra with her children. Likewise, it was emphasized by PORs that quality of life “is incredibly important”, and one representative exemplified that it can be different for a 45-year-old compared to an 85-year-old with the same disease, but also that treatments should be assessed due to the actual value they provide for patients.

### Treatment effect in relation to alternative treatments

This theme describes how a treatment’s effect can affect decisions to withdraw or withhold treatments. The healthcare service was described by both physicians and PORs as currently providing patients with ineffective treatments, and that treatments are being withdrawn too late. Participants from both groups also had some experience of treatments being both withheld and withdrawn from patients due to a lack of cost-effectiveness. However, the physicians emphasized that treatments are commonly withdrawn because they are ineffective, rather than for reasons of cost-effectiveness. The two groups also expressed that a treatment that has been proven to be ineffective for a specific patient should be withdrawn (statement 10), and this is described by the physicians as an “uncontroversial” action. One POR highlighted that she does not believe any patient wants a treatment “which is not effective, and which you do not respond to”. It was also noted by a physician that it could be an “obvious choice” to start a treatment so they can test whether it works, but that if it is not effective they must be prepared to withdraw the treatment. This view was generally supported by the PORs. Moreover, the participants expressed that it would be difficult to withdraw a treatment that had proven to be effective for a patient due to a lack of cost-effectiveness. They explained that it is preferable to not withdraw the effective treatment since they cannot be certain of how the patient will react to a treatment before it is given. One physician also stated that they “put the patient in a worse condition” if they withdraw a treatment based on economic calculations. The PORs said that the treatment should not be withdrawn, since the patient still needs the treatment and the only acceptable reason for withdrawing it is that “it doesn’t give any benefit”. These views lead to statement 12: A treatment that has proven to be effective for a specific patient should not be withdrawn by the healthcare service, even if it is not reimbursed.

“Because it always becomes a hypothetical question, because if you start a treatment where you do not know if it has a benefit for the patient, then you refrain from a treatment where you do not know the benefit, but in this patient, as you indicate, there you’ve seen a benefit for that patient, and to not get to continue for cost-effectiveness reasons, that feels much more difficult. For the other patient, it might have been that you had started a treatment and it hadn’t been effective, so that’s probably the difference, I think.” (PHY3)

Furthermore, the physicians stated that patients who have participated in clinical studies and have had access to a treatment that has proven effectiveness but has not been subsequently recommended due to a lack of cost-effectiveness should not have their treatment withdrawn. This was described as punishing a patient who has made sacrifices for the researcher by “pulling away the carpet”, and as “not ethically correct”. The PORs generally shared this view. However, one representative expressed that patients can understand if a treatment is withdrawn after a clinical study (statement 14) as “it was only research”, while another emphasized that participants in a study must be aware that studies come to an end.

The physicians highlighted that if a patient had an expected positive effect from a treatment, then they would ideally neither withdraw nor withhold it, and the opposite if a negative effect was anticipated. Similarly, both the physicians and the PORs said that it can be helpful for a physician to evaluate a treatment’s effects when deciding to withdraw a treatment (statement 16). A concern was voiced by the PORs that it can be difficult to assess a patient’s benefits from a treatment, as the current methods might “not fit so well with the diagnosis group’s perception of the severity” or possibly measure the patient’s compliance rather than the effectiveness of the treatment. The physicians also acknowledged these concerns. Finally, PORs highlighted an issue that the use of one treatment could exclude the use of alternative treatments (statement 18) due to the treatment’s side-effects, which would reduce the number of available alternative treatments if their treatment is withdrawn.

### Patient–professional communication

This theme described how communication between the patient and the physician can affect decisions to withdraw or withhold treatments. Both physicians and PORs highlighted that involving patients in decision-making can facilitate treatment withdrawals. The physicians said that if they had involved the patient throughout the treatment discussion and explained their reasons for their decision, then it became a “clear logic” for the patient. Likewise, asking the patient “What is important for you?” and reaching a consensus made the process easier. The PORs emphasized the importance of letting patients make their own decisions, as this would help treatment withdrawal.

“So, I don’t really understand why it should be so difficult to withdraw treatments and take away treatments. I believe that we overdramatize it, because it’s more about us needing to be able to talk with each other, you must be able to, and the profession must have that, so that they talk with the patients and explain it in such a way that, so the patients can also make their own choices.” (POR2)

Moreover, it was acknowledged by both physicians and PORs that agreements between a physician and a patient can facilitate treatment withdrawals (statement 20). They shared examples of agreements such as the circumstances under which the treatment will be withdrawn and how the treatment might be withdrawn in the case of a negative official recommendation. It was noted by one POR that the history of previous agreements between a physician and a patient could be the difference between withdrawing and withholding treatments, as a patient who has their treatment withheld does not have “the same history or agreements”, and it is an “isolated decision” compared to when treatment is withdrawn.

Finally, both physicians and PORs highlighted that it can be easier to withdraw a treatment if the physician informs the patient of the conditions for the treatment before starting it (statement 22). This was described by the physicians as “preparing the patient”, as “not giving any false hopes”, and as important if a treatment does not have an official recommendation and might have to be withdrawn in the future. One POR stated that “you can do a lot” if the patient is informed, and if the patient is not clearly informed and the physician wants to withdraw the treatment creates a situation “which becomes very, very difficult between the patient and the treating physician”. Likewise, both interviewed groups emphasized the importance of ensuring that the patient understands the information given to them, so that the patient does not have false belief in an ineffective treatment and instead has the right expectations.

### Patient–professional relationship

This theme described how the relationship between the patient and the physician can both facilitate and hinder treatment withdrawal. The physicians and the PORs unanimously held the view that the physician should represent the patient when deciding to withdraw or withhold treatments (statement 24). The physicians noted that the patient’s needs come first in cases where there are no recommendations, and they would not see withdrawing a treatment due to a lack of cost-effectiveness as an obligation, as the patient is—as one physician put it—“their client”. The PORs suggested that the physician should be “the patient’s best friend” and “not discuss money” with the patient.

The two interviewed groups acknowledged that having a relationship between the physician and the patient can facilitate treatment withdrawal (statement 25). The physicians described how this relationship means that they had “been there throughout the journey”, that they can win the patient’s trust, and that it became easier to get “the information needed to make a wise decision”.

“However, I should know the patient, know the patient’s needs, know the patient, have a perception of the patient’s view of their own illness and their own treatment and their own quality of life, so it’s, you must have both an outside perspective which is quite medical, risks, opportunities, purely medically.” (PHY5)

The PORs noted that it can be easier to accept a treatment withdrawal if the healthcare service offers emotional support after delivering the decision. Some shared examples such as setting aside more than 20 min for such a meeting or letting a nurse come in and explain everything again after the physician has left. One physician also highlighted that it can be comforting for relatives if the physician has the final say on withdrawing or withholding a treatment, as they can feel obliged to “fight for the child in every way”.

Physicians expressed a concern that having too close a relationship with a patient could cause them to act unprofessionally as it becomes more “emotionally charged” to withdraw a treatment and more difficult to maintain a “clear logic in the decision-making” if they have a close relationship. One physician also highlighted that having patient gratitude as a personal motivator can make physicians less inclined to withdraw treatments. However, they also expressed that professional experience makes it easier not to be affected by these emotions.

Both the physicians and the PORs highlighted that the physical meeting with patients makes it more difficult for physicians to decide to withdraw or withhold treatments for specific patients than for patient groups (statement 29). This was described by the physicians as psychologically difficult, as they “want to do what’s best for that individual” in front them and that it felt difficult to explain decisions made by “an economist without a medical background”. The PORs acknowledged the differences between making general decisions and decisions for specific patients, and one representative highlighted that in the physical meeting, the patient “has a human value, which should be equal for everyone”.

### Healthcare responsibility

This theme explains the perceived responsibilities of the healthcare system and its physicians when withdrawing and withholding treatments. The participants unanimously held the view that it is a physician’s obligation to withdraw ineffective or harmful treatments (statement 30). This obligation included discussing it with the patient and withdrawing ineffective treatments in time. The participants also identified that a physician has more obligations when prescribing unofficial treatments to patients (statement 31). One physician described that they had to be aware of going off-label, explain why they are doing so, but also know that such treatments exist and provide them to patients if needed. One POR emphasized that physicians need to know if there are any predictable risks that the treatment will not be recommended.

“In such cases it could be that if there’s a treatment which isn’t assessed, and where you don’t have any national guidelines, then it’s first and foremost the treating physician’s responsibility to know about it, and then to ensure that the patient gets it. And that can often be associated with quite a long process involving financing and so on. And that’s very different from case to case, and it’s different at different clinics, and it’s different in different regions.” (PHY7)

The physicians were also concerned that patients might lose confidence in the healthcare system if effective treatments are withdrawn because of reimbursement status (statement 32). This was described as “letting the patient down”, and taking away the patient’s hope for improvement. One physician highlighted that the patient has paid income and payroll taxes, and expects to be insured against having their effective treatment withdrawn. The PORs were generally supportive of having these expensive treatments publicly funded as it is “unsustainable” to let clinics finance treatments. There were some concerns that public funding would not solve every problem, and that current payment models need to be more like privatized models, but with the acknowledgement that clinics should be able to turn to a higher level of the healthcare system if they cannot continue to finance an expensive treatment. Moreover, the physicians expressed that the pharmaceutical company should finance effective treatments for patients after a study is completed until an official recommendation is given (statement 34).

### Ethical values

This theme describes the ethical values and their relative importance when withdrawing and withholding treatments. The physicians and the PORs expressed that there is a psychological difference between withdrawing and withholding treatments, with withdrawing being described as more difficult. The physicians generally thought there was also an ethical difference, as the physician sends an “unpleasant” message to the patient, but also that withholding can be justified since the decision would be based on “new knowledge”. The PORs emphasized that cost-effectiveness is important, but that it should be weighed against human values and the need for treatments. It would be “more immoral” to withdraw than to withhold, as giving a treatment gives the patient hope; a treatment that then would be “snatched away”.

The physicians and the PORs expressed that it is more important for physicians to make an individual assessment for patients with previous access to treatments that lack cost-effectiveness than to withdraw treatments to uphold patient equality (statement 37). The physicians acknowledged the distributive injustices caused by not withdrawing a cost-ineffective treatment, but assessed this to be a lesser problem than withdrawing a proven effective treatment. It was highlighted by one physician that patients will always get access to different treatments as time passes, and that cost-effectiveness is not a strong argument for specific patients. The PORs also acknowledged that there are difficulties associated with mixing money with decisions at an individual level, and emphasized the need for “humanity” and “being human” in human meetings. Furthermore, the representatives noted that patients might not experience the same human value if their treatments are withdrawn due to a lack of cost-effectiveness (statement 38). They also expressed that the patient would “feel pretty useless”, “sad and less valued”, “offended”, etc. One representative stated that it would be unethical to make a patient “part of a budget”, or to only look at the bigger picture and not see the human.

“You should not do it [withdraw the treatment]. I think it’s unethical that I suddenly become part of a budget. I’m never a budget. I’m a patient, and that patient should have adequate care, and it should be evidence-based, and he or she should have the best care, full stop. Because it becomes tricky then. No, we cannot afford it.” (POR5)

Furthermore, the physicians and the PORs identified that a patient who had their treatment withdrawn due to a lack of cost-effectiveness would probably seek healthcare from another healthcare provider. They shared examples of contacting another region or looking abroad. Similarly, the two interviewed groups believed that it is unjust when different healthcare providers withdraw and withhold treatments unequally (statement 40). The physicians stated that it would not be “equal care” and one physician described it as a “heavy ethical stress” which could be worse than simply not providing the treatment. The PORs described it as “horribly unequal”, causing “frustration” and a sense of “giving up”, and one criticized it for happening in Sweden, which “should be so equal and good, and democratic”.

### Professional support

This theme describes the need for and attributes of supporting tools for physicians when withdrawing and withholding treatments. The physicians described how they feel alone when having to decide whether to withdraw or withhold a treatment. This was expressed by one physician as a “vulnerable” situation for the individual physician, and that these decisions have “taken their toll” on her. The physician generally felt that it would be easier to consult a colleague when deciding to withdraw or withhold a treatment, as they would feel less vulnerable if “it isn’t the individual physician who’s made the decision”. Likewise, they highlighted that making decisions together with other colleagues would reduce the risk of the medical decision being negatively affected by the physician’s relationship with the patient. A concern was voiced by one physician that physicians can sometimes have different opinions, resulting in quite “lively discussions”.

The physicians and the PORs unanimously held the view that guidelines from a national level on treating new patients and patients with previous access to treatments after new recommendations can facilitate treatment withdrawals for physicians and patients (statement 43). The potential guidelines were described as making things more equal between regions and leaving individual physicians less vulnerable, and as a decision that politicians and the profession must take. One physician also highlighted that these guidelines need to be readily accessible for physicians so they can get a “good digital overview of what’s okay and not okay”. A concern was raised by the PORs that guidelines from a national level might not be applicable in all individual cases, and that a continuous discussion on healthcare service prioritizations is needed.

“Yeah, I mean, once again some kind of equality perspective. It becomes very complicated if we offer treatments to some, but say no to others for cost reasons. Then we find ourselves in an ethical swamp, which becomes extremely difficult for us in the healthcare service to handle. So, but it’s enormously important that the healthcare service and authorities achieve clarity in decisions concerning expensive treatments. Yeah, but when TLV carries out trials and so on, there is some sort of stringency in it all.” (PHY8)

The physicians noted that it could be helpful to have reflected on ethical problems related to priorities when making priority decisions. They explained that being involved in “provoking philosophical dialogues” and being involved in discussions about priorities could change their point of view, but also that it “felt good” and “safe” to be familiar with these thoughts.

### Reimbursement system

This theme describes reimbursement system factors which can be important for understanding the context in which decisions about withdrawing and withholding are made. The physicians and the PORs were generally positive toward healthcare making priority decisions, and explained that it is important to follow official guidelines so the healthcare service can afford future treatments and be able “to treat everyone”. They also identified a potential problem regarding priorities between patient groups as physicians tend to prioritize treatments for their own patient groups (statement 48) and patient organization representatives represent their own patient groups (statement 49). However, one representative noted that patient organizations could potentially work together with the healthcare service to help patients understand the prioritizations that are made.

Another concern from the PORs was that the treatment assessment process is not sufficiently transparent for patients (statement 50). It was suggested that patients might not find out why their treatments were withdrawn, and that authorities do not explain transparently how specific treatments were assessed or how the patients’ perspectives were represented. The PORs also emphasized that patients should be involved in the treatment assessment process to reduce “faulty prioritizations”, and to give each organization the opportunity to express themselves when a new treatment is being assessed, which one representative said could potentially make “more treatments cost-effective”.

“And I also believe when we see treatments that have a good value, and where patients really get the opportunity to explain this and show it, then we will also see that these treatments are cost-effective.” (POR2)

The PORs also stated that patients want access to new treatments (statement 52), but criticized the authorities for taking a long time to implement new treatments compared to the rest of Europe. The physicians also acknowledged that it can take a long time to implement treatments after they have been recommended. The two interviewed groups noted that patients cannot demand access to the experimental treatment if they get access to a new treatment by participating in a clinical study.

The physicians highlighted that there is a difference between what is medically best and what is practically possible when prioritizing treatments between patients (statement 55). These limitations could be based on “the tools I’m given”, staff availability, the number of hospital beds, and a need to “compromise and do the best you can in the situation”. One POR also acknowledged that there are various “bottle necks” in the current system, as “things happen, and reality is quite complex”.

## Discussion

In this article, we have explored physicians’ and patient organization representatives’ experiences and perceptions of withdrawing and withholding treatments in rationing situations due to relative scarcity. We found that physicians and PORs can experience withdrawing and withholding treatments as equally and unequally problematic or unproblematic. Hence, the findings indicate a complexity in connection with these terms and their relationships to each other. This complexity supports both equivalence and non-equivalence between withdrawing and withholding treatments, as they depend on different contextual factors. Accordingly, these factors range from individual factors, such as patients’ need for treatment and the treatment’s effects, or the relationship and communication between the physician and the patient, to more systemic factors such as healthcare responsibility, ethical values, professional support, and the reimbursement system. We also find that physicians and PORs have largely similar experiences of withdrawing and withholding treatments. Compared to previous research, our results give a more nuanced picture as there does not seem to be a clear and general answer to whether or not treatments should be withdrawn or withheld equally when rationing due to relative scarcity. Nevertheless, it is worth exploring the circumstances in which it could be justified to view the two decisions as equivalent or not equivalent.

### When withdrawing and withholding are ethically equal

Our results highlight several circumstances in which withdrawing and withholding treatments are viewed as ethically equal. The first is when a patient has an ill medical condition. In such cases, it would be easier to decide whether a treatment should be withdrawn or withheld. Likewise, having an ill medical condition also seems to increase physicians’ risk preferences and can make them less inclined to withdraw or withhold a treatment. Another circumstance is when the treatment is not effective, or is not expected to be effective, as it was viewed that such treatments should not be given to patients. Although the treatment could be (or could be expected to be) effective, it might also affect the patient’s quality of life to such an extent that the treatment should be withdrawn (or withheld). However, withdrawing and withholding treatments was viewed as equally problematic when there are few alternative treatments, which highlights that the patient’s need seems to trump any difference between withdrawing and withholding.

### When withdrawing and withholding are ethically unequal

There are also circumstances in which withdrawing and withholding are viewed as being ethically unequal. The most important factor was the prognostic difference, as both the physician and the patient can only be certain whether a treatment is effective after it has been given, which meant that withdrawing an effective treatment due to relative scarcity was viewed as more problematic than withholding a potentially effective treatment for the same reason. However, it was highlighted that it should be acceptable to withdraw treatments—and not withhold them—so that physicians can test the treatment’s potential effectiveness. This prognostic difference is also in line with previous research [17, 19]. Furthermore, the relationship between the physician and the patient can complicate treatment withdrawal, as the physician might become emotionally affected and act unprofessionally, but it can also facilitate treatment withdrawal by working as a tool to gain the patient’s trust. Moreover, the mere fact that a treatment is withdrawn implies that the patient has had previous meetings with the healthcare service and physicians, and therefore might have agreements which would distinguish the patient from new patients, further complicating treatment withdrawal. Evidently, when withdrawal and withholding are viewed as being unequal, withdrawal is commonly viewed as more problematic than withholding treatments.

### The complexity of withdrawing and withholding treatments

There seem to be potential contradictions or tensions in the results, which further complicates the issue of withdrawing and withholding due to rationing decisions. For example, the results highlight that the participants believe it is ethically more problematic to withdraw a proven effective treatment than to withdraw it to uphold distributive justice, as the human value would be disrespected. At first sight, there might seem to be an easy answer to whether treatments should be withdrawn because of financial scarcity. However, it is not clear what this valuation implies for priorities in general, as it would seem to imply a view whereby people with previous access to treatments are worth more than those without previous access (see [33, 34]). Furthermore, physicians and PORs want guidelines on withdrawing and withholding treatments due to financial scarcity at a national level so that treatments are withdrawn and withheld equally by different healthcare providers. However, they also express a need for professional discretion, as they believe that physicians should be allowed to make individual assessments about patients. In practice, it might be difficult to make these assessments equally as different physicians might have different views on what is an effective treatment, or they might have different relationships with different patients, which makes each situation unique even though the patients might have the same medical needs. Likewise, guidelines from a national level might still not be enough; although Sweden currently has national guidelines stating that unreimbursed treatments should not be used, but our interviews show that these are still used in practice. Furthermore, our results highlight a potential conflict between what is real and what is ideal. Our participants acknowledged that the healthcare service has limited resources and were supportive of medical decision makers making priority decisions. However, they also held the view that making healthcare fully publicly funded—or simply increasing the subjective value a treatment provides (which theoretically can make it cost-effective)—would be an ideal solution. Problematically, neither of these ideal solutions manages to solve the real problem of financial scarcity, as they do not consider the opportunity cost. Thus, these different conflicts further highlight the complexity of withdrawing and withholding treatments due to relative scarcity.

### Strengths and limitations

To the best of our knowledge, this is the first qualitative study on withdrawing and withholding treatments due to rationing decisions. The method is advantageous for studying experiences and perceptions, as it helps researchers to understand the underlying reasons why a phenomenon (such as preferring to withhold treatments to withdrawing them) occurs [31]. Moreover, our study is inevitably affected by the context in which it was conducted (the country, participants, researchers etc.) and hence so are the applicability, the validity, and the trustworthiness of the results. We argue that using purposive sampling helped give us a diverse and experienced sample which can reflect the experiences and perceptions of physicians and PORs. For future research, it would be of interest to further analyze withdrawing and withholding treatments due to rationing decisions by using quantitative (and experimental) methods, as the combination would make use of both methods’ strengths and counteract our potential shortcomings [35].

### Policy implications

An important implication from our results for policymakers to consider is that physicians and PORs request guidelines from a national level on withdrawing and withholding treatments due to rationing so that it is done equally between different healthcare providers. Providing treatments unequally was described as causing ethical stress (which is similar to what some scholars refer to as “moral distress”) that is worse than withdrawing treatments, which is an issue policymakers ought to consider in relation to disinvestment decisions. Furthermore, policymakers should be aware that physicians and PORs are supportive of them making rationing decisions, which is important for successful disinvestment decisions [2, 26]. Our results show that the participants views’ on access to treatment post clinical trials are in line with paragraph 34 of the WMA Declaration of Helsinki [36], patients should be granted post-trial access to beneficial treatments. The results expand on the declarations, as our participants state that the sponsors are the actor who should finance the post-trial access, until there is an official recommendation. Then, the government is suggested by our results as having the main responsibility of financing the treatment. Moreover, one solution proposed by the PORs to facilitate rationing of treatments is to involve them in the treatment assessment process and increase the process’s transparency, which could increase their supportiveness and further the external legitimacy of such decisions [37]. However, it is unclear whether this would undermine the role of experts currently making these decisions, which is ultimately an empirical question. Involving physicians in these decisions could counteract any such effects by increasing the internal legitimacy of these questions [37] and could also strengthen professional support. Ultimately, it might be difficult to formulate policies at this point, as more research is evidently needed.

Our results suggest some valuable strategies for how withdrawing and withholding treatments due to rationing decisions can be handled at a clinical level. Key aspects include continuously communicating with the patient, explaining the conditions for the treatment and how its effects will be evaluated, getting the patient’s consent to withdraw a treatment before implementing it if future access is uncertain, and using the patient–physician relationship as a tool to gain the patient’s trust. The withdrawal decision should then be taken together with other physicians to avoid the potential negative effects of having a relationship with the patient. Clinics can ease the practical difficulties associated with withdrawing treatments by implementing such routines, and will hopefully manage to respect both rationing decisions and the interests of physicians and patients.

## Conclusions

The question of withdrawing and withholding treatments in rationing situations due to relative financial scarcity is complicated. Our results indicate a more complex and nuanced stance on the matter, as the physicians and the patient organization representatives experienced withdrawing and withholding treatments as being both equal due to patients’ need for treatments, and unequal due to an inherent prognostic difference and the patient–professional relationship. Nevertheless, a plausible way to facilitate this issue is to implement clinical routines where patients must consent to future withdrawal before providing a treatment with an uncertain future reimbursement status, to ultimately ease a practically, socially, politically, and ethically difficult situation.

## Data Availability

The dataset generated and analyzed during the current study is not publicly available due to privacy but is available from the corresponding author on reasonable request.
